# New Approaches with Precision Medicine in Adult Brain Tumors

**DOI:** 10.3390/cancers14030712

**Published:** 2022-01-29

**Authors:** Annette Leibetseder, Matthias Preusser, Anna Sophie Berghoff

**Affiliations:** 1 Department of Neurology 1, Kepler University Hospital, Johannes Kepler University Linz, 4020 Linz, Austria; annette.leibetseder@kepleruniklinikum.at; 2 Department of Internal Medicine and Neurooncology, Neuromed Campus, Kepler University Hospital, 4020 Linz, Austria; 3 Division of Oncology, Department of Medicine I, Medical University of Vienna, 1090 Vienna, Austria; matthias.preusser@meduniwien.ac.at; 4 Christian Doppler Laboratory for Personalized Immunotherapy, Medical University of Vienna, 1090 Vienna, Austria

**Keywords:** precision medicine, targeted therapy, primary CNS tumors, adults, molecular markers, v-RAF murine sarcoma viral oncogene homolog B1 (BRAF), isocitrate dehydrogenase (IDH), neurotrophic tyrosine receptor kinase (NTRK)

## Abstract

**Simple Summary:**

Primary brain tumors are rare neoplasms with limited effective systemic treatment options. Recent advances in new molecular techniques have brought about novel information about molecular markers and potential targetable molecular alterations in brain tumors. Targeted therapeutic approaches are already established in several extracranial malignancies and its application is increasingly used and studied in the management of primary brain tumors. The aim of this article is to summarize the latest progress in precision medicine approaches in primary brain tumors.

**Abstract:**

Primary central nervous system (CNS) tumors represent a heterogenous group of tumors. The 2021 fifth edition of the WHO Classification of Tumors of the CNS emphasizes the advanced role of molecular diagnostics with routine implementation of molecular biomarkers in addition to histologic features in the classification of CNS tumors. Thus, novel diagnostic methods such as DNA methylome profiling are increasingly used to provide a more precise diagnostic work-up of CNS tumors. In addition to these diagnostic precision medicine advantages, molecular alterations are also addressed therapeutically with targeted therapies. Like in other tumor entities, precision medicine has therefore also arrived in the treatment of CNS malignancies as the application of targeted therapies has shown promising response rates. Nevertheless, large prospective studies are currently missing as most targeted therapies were evaluated in single arm, basket, or platform trials. In this review, we focus on the current evidence of precision medicine in the treatment of primary CNS tumors in adults. We outline the pathogenic background and prevalence of the most frequent targetable genetic alterations and summarize the existing evidence of precision medicine approaches for the treatment of primary CNS tumors.

## 1. Introduction

Precision medicine summarizes the approach to target tumor specific genetic alterations with specialized targeted treatments. Driver mutations can be targeted by specific inhibitors like tyrosine kinase inhibitors or antibodies. While specific driver mutations can be found characteristically in specific cancer entities, some targetable driver mutations are present across different entities. While targeted precision medicine approaches are well established in several extracranial solid tumors, little prospective evidence exists so far for primary central nervous system (CNS) tumors. 

The introduction of clinically relevant precision medicine options in CNS tumors is challenged by several factors. Components of the blood–brain/tumor barrier and the brain/tumor microenvironment potentially complicate the diffusion of large molecules like antibodies to the tumor site. Spatial and temporal heterogeneity of the tumor must be considered, especially in terms of resistance to targeted therapy, as genomic alterations and gene expression patterns differ spatially within the tumor or may change over time [1,2]. Further, most targetable genetic alternations are only infrequently observed in CNS tumors. Therefore, designing clinical trials to investigate the clinical efficacy is challenging.

Nevertheless, in the past decade, several targeted therapies were evaluated in phase II and phase III trials in brain tumors. Prospective studies concentrated on programmed death-1 (PD-1) inhibitors [3,4,5,6], integrin inhibitors [7], anti-angiogenic therapies targeting vascular endothelial growth factor (VEGF) [8,9,10] or VEGF receptors (VEGFR) [11,12,13], targeted treatment against epidermal growth factor receptor (EGFR) [14], and inhibition of cyclin dependent kinases (CDK) [15,16,17].

In the following we give a short overview of performed phase II and III studies and their results investigating the abovementioned targets:

(a) PD-1 inhibitors:

Two randomized, multicenter phase III trials investigated the relevance of adding Nivolumab to standard of care treatment in the first-line setting of MGMT-unmethylated (Checkmate 498; nivolumab plus radiation versus temozolomide and radiation) and MGMT-methylated (Checkmate 548; nivolumab plus temozolomide and radiation therapy versus placebo plus standard of care) glioblastoma patients [4,5].

An open label randomized phase III clinical study (Checkmate 143) evaluated outcome after PD-1 inhibition with nivolumab compared to bevacizumab in glioblastoma patients at first recurrence [3]. 

All of these studies did not meet their primary endpoint, showing that PD-1 immune checkpoint inhibition treatment did not improve overall survival (OS) in glioblastoma patients, neither at initial diagnosis nor at recurrence [3,4,5].

Pembrolizumab represents another PD-1 inhibitor which has been investigated in phase I/II trials in primary brain tumors. A multicohort phase I trial in which 26 recurrent glioblastoma patients received treatment with pembrolizumab demonstrated response rates of 8% (95% confidence interval (CI), 1–26%) [18]. A recently published phase II study randomized 80 glioblastoma patients with recurrent disease to pembrolizumab with bevacizumab or pembrolizumab monotherapy. In this study, no impact on progression free survival (PFS) of pembrolizumab as monotherapy or in combination with bevacizumab was observed [6].

(b) Integrin inhibitors:

Cilengitide showed no outcome benefit in a multicenter randomized, open-label phase III trial as a combination with temozolomide chemoradiotherapy in newly diagnosed glioblastoma [7].

(c) VEGF and VEGFR inhibitors:

The effectiveness of bevacizumab, a humanized monoclonal antibody for VEGF-A, was extensively studied either as a single agent or in combination with other therapies in newly diagnosed and progressive glioma [8,9,10,19,20,21,22,23,24]. However, phase III studies failed their primary endpoint in showing improvement of OS, but demonstrated PFS benefit [9,10]. 

Sunitinib malate is a small-molecule tyrosine kinase inhibitor (TKI) targeting VEGFR and platelet-derived growth factor receptor (PDGFR), and shows insufficient activity in phase II trials as a monotherapy or combined with irinotecan in recurrent glioma [11,12,20,25]. 

Cediranib represents an oral pan–VEGF receptor TKI. Its efficacy was evaluated in a phase II trial [26] and thereafter in a randomized, phase III, placebo-controlled, partially blinded clinical trial in recurrent glioblastoma (cediranib as monotherapy or in combination with lomustine versus lomustine). The study did not meet its primary endpoint of PFS prolongation [13].

(d) EGFR inhibition:

Many approaches to target EGFR have been developed, including therapy with small molecule TKIs (for example, gefitinib and lapatinib), monoclonal antibodies (cetuximab, nimotuzumab), antibody drug conjugates (depatuxizumab mafodotin) or vaccination with rindopepimut. None of these agents demonstrated outcome benefit in primary brain tumors [14,27,28,29,30,31].

(e) CDK inhibitors:

The cyclin D-CDK4/6-Rb pathway is dysregulated in many cancer types. Therefore, inhibition of this pathway has emerged as a promising target for cancer treatment [32,33]. As an example, cyclin-dependent kinase 4/6 (CDK4/6) inhibitors including palbociclib, abemaciclib and ribociclib are well established therapies in combination with endocrine therapy in breast cancer [34,35,36,37]. 

Far less data and no phase III trials exist in adult primary brain tumors. A phase II study from the Spanish group for Research in Neuro-Oncology was conducted to evaluate the efficacy of palbociclib monotherapy in recurrent Retinoblastoma-positive anaplastic oligodendroglioma. In this study with inclusion of 34 patients, no PFS benefit was observed. There were no partial or complete responses, and 13/34 (38%) patients achieved stable disease [17]. 

Another phase II trial did not show antitumor activity of palbociclib in adult patients with recurrent RB1-positive glioblastoma and was prematurely terminated [15]. 

Recently, impact on OS of abemaciclib therapy in newly diagnosed glioblastoma compared to a control group was evaluated in a phase II ‘platform trial’ (Individualized Screening Trial of Innovative Glioblastoma Therapy—INSIGhT). Patients in the abemaciclib arm demonstrated no significant improvement on OS, but good drug tolerability and an increase in PFS [16]. 

Zotiraciclib (TG02), a potent CDK9 inhibitor, is currently investigated in recurrent and in newly diagnosed high-grade glioma (NCT02942264, NCT03224104).

In summary, none of the studies resulted in a clinically meaningful prolongation of the survival time, and in consequence, none of these agents entered clinical practice. Nevertheless, there are several promising targets, which are currently evaluated in preclinical studies. Here, agents targeting within oncogenic pathways like Kirsten rat sarcoma 2 viral oncogene homolog (KRAS), mesenchymal-epithelial transition factor (MET), telomerase reverse transcriptase (TERT) and alpha-thalassemia/mental retardation syndrome X-linked (ATRX), are currently under investigation [38,39,40,41] and might move forward to clinical application in the next years. 

In the following review, we mainly concentrate on new and promising clinical targeted therapy approaches in adults. Latest advances of targeted treatment options in the clinical management of pediatric CNS tumors were recently reviewed elsewhere [42,43].

In this review, we summarize the current evidence on targeted therapies with focus on v-RAF Murine Sarcoma Viral Oncogene Homolog B1 (BRAF), isocitrate dehydrogenase 1 (IDH) and neurotrophic tyrosine receptor kinase (NTRK) fusion in a precision medicine approach for adult primary brain tumors. 

## 2. Review

### 2.1. v-RAF Murine Sarcoma Viral Oncogene Homolog B1 (BRAF) Inhibitors 

The v-RAF Murine Sarcoma Viral Oncogene Homolog B1 (BRAF) gene encodes for rapidly accelerating fibrosarcoma (RAF) serine/threonine kinases. In normal cells, BRAF is activated by extracellular growth signals whereupon signals of rat sarcoma protein (RAS) are transduced downstream via the mitogen activated protein kinase signaling pathway (MAPK) [44,45]. BRAF alterations lead to constitutive MAPK signaling pathway activation, bypassing the need of proliferative signals. Thereby, cellular proliferation, survival and dedifferentiation are promoted either through their kinase activity, RAS dependency, or dimerization status [46,47]. Activating mutations of BRAF occur as a point mutation, in-frame deletion, or fusions with other kinases. The BRAF V600E point mutation (c.1799T > A) leads to a substitution from valine to glutamic acid at position 600, and is the most common one in a subset of CNS tumors [48,49] and other cancers [50,51,52]. 

BRAF is known as a common mutated kinase in various human cancer types [46,50,53] including melanoma [50,54], colorectal cancer [55], thyroid cancer [56], non-small-cell lung cancer (NSCLC) [57,58], hairy cell leukemia [59], and primary CNS tumors [48].

**Figure 1:**
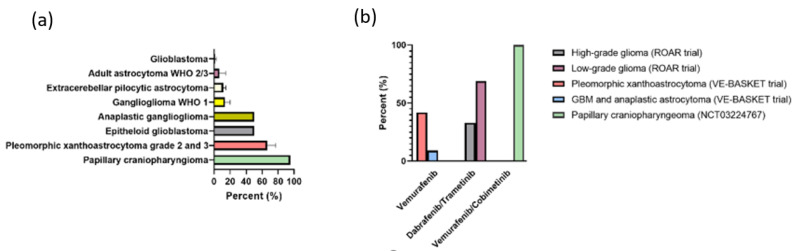
(**a**) Prevalence of v-RAF Murine Sarcoma Viral Oncogene Homolog B1 (BRAF) V600E mutation in papillary craniopharyngioma, pleomorphic xanthoastrocytoma, ganglioglioma, glioblastoma, pilocytic astrocytoma and adult astrocytoma World Health Organization (WHO) 2/3; (**b**) Response rates of vemurafenib monotherapy (VE-BASKET study), combined therapy with dabrafenib/trametinib in low- and high-grade glioma (NCT02034110) and vemurafenib/cobimetinib in papillary craniopharyngioma (NCT03224767).

Frequency rates of BRAF V600E mutation in primary CNS tumors are illustrated in Figure 1a. It is frequently present in papillary craniopharyngioma (95%) [60] and in two thirds of WHO grade II or III pleomorphic xanthoastrocytoma (PXA) [48]. Additionally, BRAF V600E mutation is encountered in WHO grade I ganglioglioma (GG), anaplastic GG, and extracerebellar pilocytic astrocytoma (PA I) in up to 18%, 50%, and 9%, respectively [48]. Among glioblastoma (GBM), BRAF V600E mutation can be found in 1–8% of patients [48,49,61], whereas a higher mutation rate is shown in patients below the age of 30 years (20%) [49,61]. The subtype of epithelioid GBM harbors BRAF V600E mutation in approximately 50% of the cases [62,63]. 

The occurrence of the oncogenic KIAA1549-BRAF fusion is characteristic of cerebellar PA I in pediatrics and less frequent in extra-cerebellar PA I [64,65,66]; however, it has different biological consequences as the BRAFV600E point mutation. The efficacy of the currently available BRAF inhibitors is limited to point mutations including the BRAF V600E. According to preclinical studies and data in various cancers, tumors carrying a BRAF fusion are not sensitive to BRAF inhibitor therapy [67]. Further, resistance to BRAF inhibition was observed in V600E-mutated glioma cell lines with additional epidermal growth factor receptor (EGFR) amplification [68].

Vemurafenib and dabrafenib function as selective oral inhibitors of the BRAF V600E kinase. In patients with cerebral metastatic melanoma, higher response rates were observed by usage of dabrafenib compared to vemurafenib (31% versus 16%, respectively) [69,70]. A reason for it might be better mobility of dabrafenib through the blood brain barrier (BBB) due to its smaller size and different molecular structure [71]. 

Tumors’ resistance against RAF inhibitor monotherapy frequently occurs due to maintenance of MAPK pathway activation [72] and prompted the recommendation of adding a MEK inhibitor in order to set another blockade. The efficacy of combined treatment with MEK-inhibitor including dabrafenib/trametinib, was approved in melanoma [73] and NSCLC [74]; the combination of vemurafenib/cobimetinib and encorafenib/binimetanib only in melanoma [73,75,76]. 

Far less clinical evidence exists in primary CNS tumors. Experiences are based on case reports/series in the recurrent setting [77,78,79,80,81] and phase II trials. Findings with corresponding response rates to BRAF monotherapy or combination therapy with MEK inhibitors in brain tumors are shown in Figure 1b. 

In an open-label, nonrandomized multicohort, ‘basket’ study (VE-BASKET) efficacy and safety of vemurafenib monotherapy was shown in patients with nonmelanoma tumor including 24 BRAF-V600E mutated gliomas [82]. The glioma subgroup in this study showed an overall response rate of 25% [82]. The response rate of GBM and anaplastic astrocytoma was 9%, but was more encouraging in pleomorphic xanthoastrocytomas with a rate of 42% [82]. 

Recently published interim results from another ongoing open-label, single-arm, phase 2 basket trial (Rare Oncology Agnostic Research—ROAR study, NCT02034110) evaluating combined dabrafenib/trametinib in adults with recurrent high-grade (*n* = 45) or low-grade glioma (*n* = 13) showed response rates of 33% (15/45, CI 95% 20–49) and 69% (9/13, CI 95% 39–91), respectively [83].

Findings from a phase II study of therapy-naïve papillary craniopharyngioma patients treated with oral vemurafenib and cobimetinib demonstrated an objective response in all patients (16/16, 100%). A second arm of this study is currently recruiting patients with progressive papillary craniopharyngiomas after radiotherapy [84]. 

In adults, a phase II clinical trial examining the efficacy of combination treatment with encorafenib and binimetinib in recurrent BRAF V600E-mutated high-grade glioma (HGG) and PXAs (NCT03973918) is ongoing. In pediatrics, several more phase II clinical trials examining BRAF and/or MEK inhibition are underway (NCT04201457, NCT01748149, NCT04775485, NCT02684058, NCT03363217).

So far, no evidence regarding BRAF inhibition as first-line treatment in primary CNS tumors exists. However, a currently recruiting phase II study (NCT03919071) is evaluating the benefit of usage of dabrafenib with trametinib after local radiotherapy in newly diagnosed BRAF V600-mutant HGG in pediatrics and young adults.

### 2.2. Isocitrate Dehydrogenase 1 (IDH1) Inhibition

Mutations in the gene encoding isocitrate dehydrogenase 1 (IDH1) and 2 (IDH2) were identified across several cancer types including gliomas, chondrosarcoma, and hematological malignancies [85,86,87,88,89,90]. IDH1 or two mutations are known as an early and significant contributor to tumorigenesis by intracellular accumulation of an oncometabolite product called 2-hydroxyglutarate (2HG) [91]. The latter drives downstream metabolic changes and alters epigenetic and genetic profiles, resulting in genome-wide CpG island hypermethylation, increased repressive histone methylation and double-strand DNA breaking [92,93,94,95,96,97].

**Figure 2:**
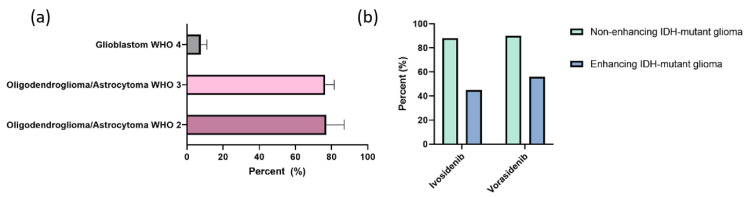
(**a**) Prevalence of Isocitrate Dehydrogenase (IDH) mutation in gliomas; (**b**) disease control rate of IDH-inhibitors in contrast-enhancing and non-contrast enhancing IDH-mutated glioma.

A point mutation in IDH1 at the codon 132 (IDH1 R132H), resulting in a switch from arginine to histidine, represents the most common IDH mutation in gliomas [86]. It is seen in approximately 80% of WHO grade II/III gliomas [86,98,99] and in 4–12% of GBM [87,90,99], whereas only a minority of gliomas (4–8%) harbor an IDH2 mutation [86,98,100] (Figure 2a). IDH1 or 2 mutations also occur in other malignancies, including chondrosarcoma [85], intrahepatic cholangiocarcinoma [101], and acute myeloid leukemia (AML) [88,102]. 

IDH inhibitors are established therapy options in AML with overall response rates of 38.8% to enasidinib and 41.6% to ivosidenib (AG-120) [103,104]. The role of IDH inhibitors in solid tumors including glioma is currently under investigation. A phase I study, followed by a randomized, placebo-controlled phase III trial in advanced IDH1-mutated cholangiocarcinoma reported prolonged progression-free survival (PFS) and good tolerability of ivosidenib [105,106]. 

In IDH1 mutated glioma, a multicenter open-label phase I dose escalation study of ivosidenib showed a favorable safety profile as well. Regarding outcome, nonenhanced tumors in this cohort showed an objective response rate of 2.9% and a prolonged median PFS rate with 13.6 months (95% CI, 9.2–33.2 months), whereas the median PFS of gliomas with enhancing lesions was 1.4 months (95% CI, 1.0–1.9 months) [107]. Stable disease was achieved in 30 of 35 non-enhancing glioma patients (85.7%) in comparison to 14 of 31 enhancing glioma patients (45.2%). These data were supported by the results of a succeeding perioperative trial, which showed considerable reduction of intratumoral 2-HG levels by 90% in non-enhancing recurrent IDH1 mutant low-grade glioma [108]. DS-1001b is another oral selective inhibitor of mutant IDH1 R132X. Its safety, brain distribution, and treatment response has been investigated in recurrent glioma within a phase I trial (NCT03030066) [109]. Final results are not published yet, but preliminary analysis showed a favorable safety profile and clinical responses [109]. BAY 1436032 was developed as a pan-inhibitor of IDH1 protein with different codon 132 mutations. In murine orthotopic xenograft models, a reduction of intratumoral 2-HG levels and prolonged survival was detected [110]. These preclinical data led to the design and initiation of a phase I clinical trial in patients with IDH1-mutated advanced solid cancers (NCT02746081). 

The oral drug vorasidenib (AG-881) represents a novel pan-inhibitor of mutant IDH1 and IDH2. Dual inhibition of mIDH1/2 may be of advantage in the case of occurrence of isoform switching from mIDH1 to mIDH2 or conversely, which has been noted as a potential acquired resistance mechanism against isoform-selective inhibitors in AML [111]. Preclinical trials using vorasidenib in orthoptic mouse xenograft models of human IDH1/2 mutated glioma demonstrated improved BBB penetration, significant 2-HG suppression, and inhibition of tumor growth [112,113]. Data of a multicenter, single-arm phase I dose-escalation study of AG-881, which enrolled 52 patients with recurrent or progressive glioma, were recently published [114]. Results of this study highlighted good drug tolerability with the exception of reversible elevated transaminases as dose-limiting toxicity (≥100 mg). 

Similar to the findings with ivosidenib [107], antitumor activity in this cohort was restricted to patients with non-enhancing glioma. Objective response rate was 18.2% (95% CI, 5.2–40.3) in non-enhancing tumors and 0% (95% CI, 0–11.6) in enhancing glioma. Median PFS and disease control rate was 36.8 months (95% CI, 11.2–40.8) and 90.8%, respectively, whereas enhanced tumors showed no responses, but stable disease in 17/30 (56.7%) and a median PFS of 3.6 months (95% CI, 1.8–6.5) [114]. 

Observed disease control rates to IDH-inhibitors in gliomas of the just mentioned phase I trials are demonstrated in Figure 2b.

Based on data of the abovementioned previous studies, a randomized, placebo-controlled phase III trial has been designed with the aim to investigate the efficacy of AG-881 in patients with residual or recurrent non-enhancing IDH1 or 2 mutated grade 2 glioma (INDIGO-trial, NCT04164901). 

Another personalized treatment approach is to target IDH1(R132H)-mutated malignancies via IDH1(R132H)-specific peptide vaccines (IDH1-vac) [115,116]. 

IDH1(R132H) functions as a driver mutation and generates immunogenic neoepitopes which are presented on major histocompatibility complexes (MHC) to stimulate T cell responses. Preclinical studies in MHC-humanized mice provided evidence to control syngeneic IDH1(R132H)-expressing tumors by inducing a mutation-specific T-helper cell response [117,118]. Based on these data, IDH1(R132H)-specific peptide vaccines have found entrance into clinical phase I trials. 

A first-in-humans, multicenter, single-arm open-label phase I trial (NOA-16, NCT02454634), which included 33 patients with newly diagnosed WHO grade 3 and 4 IDH1(R132H) mutated astrocytoma, demonstrated safety and immunogenicity of IDH1-vac [115]. 

By now, two more different IDH1-directed mutation-specific peptide vaccines have been developed (PEPIDH1M, IDH1R132H-DC vaccine) and are currently tested in two ongoing single-arm phase 1 trials (NCT02193347, NCT02771301). 

The currently recruiting NOA-21 trial (NCT03893903) aims to assess safety and efficacy of combining IDH1-vac with the programmed death-ligand 1 (PD-L1) inhibitor Avelumab in recurrent glioma. The study is designed as a randomized phase I trial with three treatment arms (Arm 1: IDH1-vac, Arm 2: IDH1-vac + Avelumab, Arm 3: Avelumab).

### 2.3. Neurotrophic Tyrosine Receptor Kinase (NTRK) Fusion Inhibition 

Three neurotrophic tyrosine receptor kinase (NTRK) genes, NTRK1, NTRK2 and NTRK3, encode for the respective transmembrane tyrosine-kinase receptors (TRK-A, TRK-B, TRK-C) [119,120,121]. Physiologically, TRKs play an important role for the regulation of neuronal differentiation and function pathways, especially during the period of embryo development [122,123,124,125]. Chromosomal rearrangements resulting in somatic gene fusions mostly occur between the 3′-end of a NTRK gene and 5’-end of another gene [126]. NTRK fusions may function as oncogenic drivers by inducing ligand-independent chimeric rearrangements in TRKs. This leads to an uncontrolled and constitutive TRK signaling activation and thus to the upregulation of several downstream pathways [120,124,127]. 

Overall, NTRK fusions are scarce and occur both in adults and pediatrics with an estimated prevalence of less than 1% across a broad range of different tumor types [120,126,128,129,130,131,132]. However, presence of NTRK fusions varies among histologic subtypes. NTRK fusions are detected at high frequencies (>90%) in distinct rare neoplasms such as breast secretory carcinomas, mammary analogue secretory carcinoma of the salivary glands, infantile fibrosarcoma, and congenital mesoblastic nephroma, and are thereby considered as pathognomonic in these tumor entities [120,133,134,135,136,137,138,139]. 

**Figure 3:**
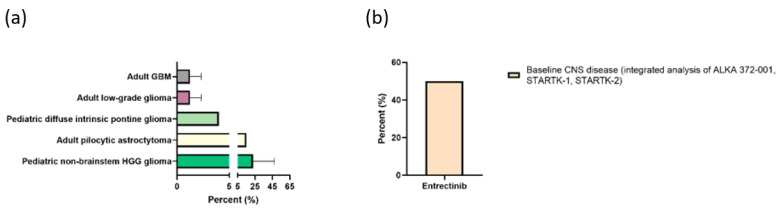
(**a**) Prevalence of Neurotrophic Tyrosine Receptor Kinase (NTRK) fusions in adult and paediatric gliomas; (**b**) response rates of entrectinib monotherapy (ALKA 372-001, STARTK-1; STARTK-2, NCT02650401).

Regarding CNS tumors, NTRK fusions are present in approximately 0.55 to 2% of glioma and neuroepithelial tumors [129,130,140,141,142]. Prevalence of NTRK fusions in different tumor types are illustrated in Figure 3a. Adult IDH wildtype GBM harbor NTRK gene fusion in 1–2% [140], mostly involving the NTRK2 gene and less frequently the NTRK1 or NTRK3 gene [132,140,143,144,145]. Higher prevalence rates of NTRK fusions are found in pediatrics, especially in pediatric high-grade glioma (HGG), diffuse intrinsic pontine glioma, and non-brainstem HGG patients younger than three-years-old, with 5.3%, 4%, and 40%, respectively [129,146,147,148,149,150] (Figure 3a). Moreover, NTRK rearrangements have been detected, albeit very seldomly, in several other primary CNS tumors, including pilocytic astrocytoma [140,151,152,153], ganglioglioma [153,154,155], diffuse midline glioma [156], pleomorphic xanthoastrocytoma [147], CNS fibroblastic tumor [157], and CNS embryonal tumor [158]. A recently published multi-institutional study by Torre et al. demonstrated a high diversity of clinicopathologic and molecular features of NTRK-fused gliomas with considerable differences by age group and co-occurring genetic alterations [159]. 

NTRK-fusion positive cancers represent a molecularly defined subset of tumors susceptible to targeted therapy with TKIs, regardless of tumor histology or age. Two small-molecule, orally-administered TKI-labeled medications, larotrectinib and entrectinib, obtained approval from the FDA after showing a favorable tolerability profile and high efficacy in patients with tumors harboring NTRK fusions [160,161,162,163,164,165,166,167]. By now, larotrectinib has been approved by the European Medicines Agency (EMA) in that regard as well. 

Larotrectinib acts as a selective pan-TRK inhibitor. Its efficacy and safety is/was basically studied within three clinical trials including a phase I–II trial in pediatrics trial (SCOUT, NCT02637687, still recruiting), a phase II basket trial in adults and adolescents (NAVIGATE, NCT02576431, still recruiting), and an adult phase I trial [166,168]. A pooled analysis of these studies which enrolled a total of 159 patients with advanced or metastatic non-CNS primary NTRK-fusion positive solid tumors demonstrated response rates after treatment with larotrectinib of >75%. Median duration of response (DoR) and median PFS was declared with 35.2 months and 28.2 months, respectively. The tolerability profile in 260 patients was considered as favorable through occurrence of almost mild and transient adverse events (AEs). Drug-related AEs led to treatment discontinuation in 2% and dose reduction in 8% of cases. Outcome data of enrolled patients with primary CNS tumors in these studies are still pending [168]. 

Similarly, antitumor activity and tolerability of entrectinib, a selective pan-TRK, ROS1 and ALK inhibitor have been evaluated in three clinical trials (completed phase I study ALKA 372-001, completed phase I study STARTK-1, ongoing phase II study STARTK-2 NCT02650401). An integrated analysis of these studies with a total of 54 patients revealed response rates of 59.3% (Figure 3b), a median DoR of 12.9 months and a median PFS of 11.8 months [163]. In this study cohort, entrectinib induced responses in six (50%) of the twelve patients with baseline CNS disease [163]. 

Case series/reports or subgroup analysis in previous studies revealed intracranial responses to both larotrectinib or entrectinib in brain metastasis [163,165,166,169,170] and primary CNS tumors [153,154,169,171,172,173,174,175]. 

The results of ongoing studies enrolling patients with primary CNS tumors and NTRK-fusions (for example, NCT02576431, NCT04655404, NCT02637687, NCT03213704, NCT04879121, NCT04142437, NCT03834961, NCT02650401) are awaited to provide more evidence to support the use of TKI as targeted treatment in brain tumors. 

## 3. Conclusions

Targeted therapies have shown promising response rates in selected patients with primary CNS tumors. Overall, the prevalence of the currently targetable mutations in primary brain tumors is low. Here, the main challenge is to identify patients for a potential precision medicine approach as the targetable driver mutations are not regularly assessed in every single patient. New molecular diagnostic tools like methylation analysis might allow, in addition to a precise diagnostic work up, the early identification of targetable mutations. 

The clinical evidence for targeted treatment approaches in primary CNS tumors is limited by the, so far, low number of treated patients in a prospective manner. Nevertheless, modern trial design in precision medicine, particularly including ‘basket and platform trials’, focuses on the further development of targeted treatments in patients with primary CNS tumors. 

‘Basket trials’ allow inclusion of patients presenting with a specific driver mutation irrespective of the underlying tumor type. In ‘platform trials’, patients are screened for a broad range of driver mutations and assigned to a particular treatment based on the results of the genetic testing. 

In the field of precision medicine, three relevant open questions are warranted to be integrated into future studies. First, when is the optimal treatment start of these drugs (at initial diagnosis or at recurrence)? Second, what is the role of combining molecular treatment with radiotherapy? Finally, how do these agents influence patient-related outcome, especially quality of life?
